# Mesenchymal Stromal Cells Perspective: New Potential Therapeutic for the Treatment of Neurological Diseases

**DOI:** 10.3390/pharmaceutics13081159

**Published:** 2021-07-27

**Authors:** Takeo Mukai, Kenshi Sei, Tokiko Nagamura-Inoue

**Affiliations:** 1 Department of Pediatrics, The University of Tokyo Hospital, Hongo, Bunkyo-ku, Tokyo 113-8655, Japan; 2 Department of Cell Processing and Transfusion, The Institute of Medical Science, The University of Tokyo, 4-6-1 Shirokanedai, Minato-ku, Tokyo 108-8639, Japan; k-sei@ims.u-tokyo.ac.jp (K.S.); tokikoni@ims.u-tokyo.ac.jp (T.N.-I.)

**Keywords:** mesenchymal stromal cell, umbilical cord, neurological disorders, cerebral palsy, neurotrophic factor

## Abstract

Several studies have shown that mesenchymal stromal/stem cells (MSCs) exert their neuroprotective and neurorestorative efficacy via the secretion of neurotrophic factors. Based on these studies, many clinical trials using MSCs for the treatment of neurological disorders have been conducted, and results regarding their feasibility and efficacy have been reported. The present review aims to highlight the characteristics and basic research regarding the role of MSCs in neurological disease and to discuss the recent progress in clinical trials using MSCs to treat various neurological disorders.

## 1. Introduction

Mesenchymal stromal/stem cells (MSCs) have been the focus of new cell therapy development due to their potential to treat neurological disorders [1]. MSCs were first discussed in 1991 when they were introduced by Caplan as mesenchymal cells in bone marrow [2]. Now, MSCs have been isolated from several sources, including bone marrow (BM), the umbilical cord (UC), umbilical cord blood (UCB), dental pulp (DP), and adipose tissue (AD) [1,3,4,5,6,7,8].

**Figure 1:**
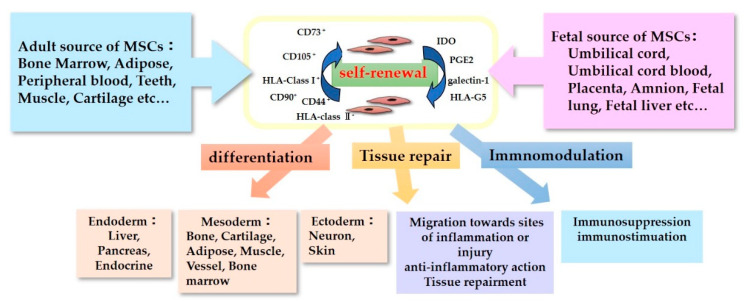
Characteristics of Mesenchymal stromal/stem cells (MSCs).

The characteristics of MSCs are defined by a set of criteria that form the basis for their clinical use (Figure 1). The International Society for Cellular Therapy proposed the criteria for defining human MSCs [9,10]. Firstly, the MSCs must be plastic-adherent when maintained in standard culture conditions. Secondly, they must express CD105, CD73, and CD90, but not CD45, CD34, CD14 or CD11b, CD79α or CD19, and HLA-DR surface molecules. Thirdly, MSCs must be able to differentiate into adipocytes, chondroblasts, and osteoblasts in vitro. Immunomodulatory effects are the most important and popular property of MSCs in their clinical use [11]. MSCs lack HLA-class II expression and do not express the co-stimulatory surface antigens CD80 or CD86, which activate T-cells [12]. As a result, these cells are able to escape from activated T-cells. MSC-mediated immunomodulation results from the MSC secretome, which includes components such as indoleamine 2,3-dioxygenase (IDO), PGE2, galectin-1, and HLA-G5 [13]. With these anti-inflammatory properties, MSCs could be useful therapeutic candidates for use in the treatment of neurological disorders accompanying inflammation.

The tissue repair properties of MSCs are also important to their neurorestorative effect. The neurorestorative and neuroprotective effects of MCSs regarding tissue repair can be divided into two main mechanisms: (1) neurogenic differentiation and eternal cell replacement and (2) the secretion of neurotrophic factors [14]. Regarding the former, we have observed in our experiments that MSCs do not engraft and differentiate into neural cells, and they disappear within two weeks of administration in non-immunocompromised mouse models [15]. In contrast, we found that UC-MSCs that secrete neurotrophic factors such as brain-derived neurotrophic factor (BDNF) and hepatocyte growth factor (HGF) but not nerve growth factor (NGF) attenuate brain injury [15,16]. This MSC paracrine effect is expected to contribute toward their use in therapeutics for neurological injuries. Many studies using neurological disorder models have reported improvements in the studied conditions after the administration of MSCs, and clinical studies using MSCs to treat neurological disorders have been already conducted. 

In this review, we discuss the effects demonstrated by MSCs in neurological injury models and their mechanisms. We also summarize the recent progress in regenerative therapies using MSCs for the potential treatment of various neurological disorders.

## 2. Mechanisms of ACTION of MSC on Neurological Injury Models

MSCs have been reported to secrete heterogeneous lipid bilayer vesicles called extracellular vesicles (EVs), which act as mediators for inter-cell communication [17]. These exosomes/EVs secreted from MSCs are known to improve neuronal functions in neurological injured models [17,18]. We have also demonstrated the amelioration of neuronal injury followed by functional improvement in MSC-administered mice models, which resulted from the secretion of trophic factors rather than neuronal differentiation and eternal cell replacement by MSCs [15]. In addition, immunomodulatory effects lacking HLA-class II expression and secreting IDO, PGE2, galectin-1, and HLA-G5 are the most important property of MSCs in their clinical use. Therefore, MSCs could be useful therapeutics for neurological disorders accompanying inflammation.

Regarding the way of administration of MSCs, most studies adopt intravenous injection (IV) or intrathecal injection (IT). On the other hand, there are some notable basic research studies reporting the efficacy of MSCs by intranasal administration [19] and either stereotaxically into the striatum or intra-arterially administration through the internal carotid artery [20]. Furthermore, Leong et al. investigated three different methods: direct stereotaxic injection into the lateral cerebral ventricle, intra-carotid administration, and femoral venous infusion, and of the three different methods of MSC transplantation tested in the present study, direct stereotaxic injection induced the highest concentration of MSCs in brain areas, resulting in the best neurological outcome [21].

EVs of MSCs have been reported to promote neurite development in middle cerebral artery (MCA) stroke models [22]. Moreover, MSC-EVs significantly upregulated p-AMPK and downregulated p-JAK2, p-STAT3, and p-NF-κB, which resulted in improvements in pathological lesions in cortical brain tissue and the attenuation of neuronal apoptosis in the cortex of an MCA occlusion rat model [23]. In addition, anti-inflammatory and immunomodulatory effects of MSC-derived exosomes are reported to be exerted by miR-30d inhibiting autophagy-mediated microglial polarization to M1 [24]. On the other hand, the efficacy of dental pulp stem cells (DPSCs) for focal cerebral ischemia has been reported [25]. They demonstrated that treatment with DPSCs combined with BDNF promoted the recovery of neurological function more effectively compared with BDNF injection or DPSC transplantation alone. Furthermore, Wu et al. reported that periodontal ligament stem cells (PDLSCs) transplantation promoted the recovery of neurological function more effectively than DPSC transplantation in rats with transient occlusion of the right middle cerebral artery [26].

MSCs have also shown potential in the healing of spinal cord injury (SCI) in a rat model through inhibition of pericyte migration. This improved motor functioning and the structural integrity of the blood–spinal cord barrier [27]. Treatment with BM-MSC derived exosome have been reported to reduce neuronal cell death, improve myelin arrangement and reduce myelin loss, increase pericyte/endothelial cell coverage on the vascular wall, decrease blood–spinal cord barrier leakage, reduce caspase 1 expression, inhibit interleukin-1β release, and accelerate locomotor functional recovery in rats with SCI [28]. Moreover, Li et al. demonstrated the inhibition of neuronal apoptosis via activation of the Wnt/beta-catenin signaling pathway by MSCs in an SCI model [29].

Furthermore, hypoxic ischemic (HI) models have often been used as a mimic model of cerebral palsy. Sisa et al. reported that EVs from MSCs could exert a neuroprotective effect in mouse models with severe HI-induced neonatal brain injury [30]. Thomi et al. reported that exosomes from UC-MSCs also reached the brain and reduced microglia-mediated neuroinflammation in rats with perinatal brain injury [31]. MSC treatment is also reported to ameliorate inflammation-induced neuronal cellular degeneration, reduce microgliosis, and prevent reactive astrogliosis, resulting in improved long-lasting cognitive functions [32].

Regarding amyotrophic lateral sclerosis (ALS), the repeated administration of AD-MSC exosomes was reported to improve the motor performance; protect lumbar motoneurons, the neuromuscular junction, and muscles; and decrease the activation of glial cells in ALS model mice [33]. Corti et al. reported that BM-MSCs promoted the survival of motor neurons and improved neuromuscular function in SOD1 G93A mice, leading to prolonged disease duration and lifespan [34]. Moreover, they showed transplanted cells were engrafted within the host spinal cord. Consistently, the intravenous administration of AD-MSCs in superoxide-dismutase 1 (SOD1)-mutant transgenic mice was able to promote neuroprotective and neuroregenerative actions [35]. They also showed the migration of AD-MSCs in the anterior horn of the spinal cord, though they did not show neuronal or glial markers.

As for multiple sclerosis (MS), in vitro data demonstrated that MSC-secreted EVs promote myelin regeneration by inducing the differentiation of endogenous oligodendrocyte precursor cells into mature myelinating oligodendrocytes [36]. On the other hand, Riazifar et al. found that the intravenous administration of MSCs-EV stimulated by IFNγ reduced the mean clinical score of encephalomyelitis mice compared to PBS control, reduced demyelination, decreased neuroinflammation, and upregulated the number of CD4+CD25+FOXP3+ regulatory T cells (Tregs) within the spinal cords of encephalomyelitis mice [37].

Parkinson’s disease is a common neurodegenerative disorder, and effective treatments are not available at the present. Choi et al. reported that the intravenous administration of AD-MSCs significantly improved the behavioral performances at 3 weeks after the injection of MSCs in the Parkinson’s disease mouse model induced by 6-hydroxydopamine. Additionally, dopaminergic neurons were rescued, the number of structure-modified mitochondria was decreased, and the mitochondrial complex I activity was restored in the brains of the AD-MSCs injected mouse, which suggests that AD-MSCs may have therapeutic potential for Parkinson’s disease by recovering mitochondrial functions [38]. On the other hand, a Rotenone-induced rat model is also used for Parkinson’s disease mouse model. The intravenous administration of BM-MSCs was able to migrate to the injured brain, and they significantly decreased serum TGF-β1 levels and increased levels of serum BDNF and brain dopamine [39].

There are also some reports showing the efficacy of MSCs for traumatic brain injury models. Compared with the vehicle, human BM-MSCs exosome treatment significantly improved sensorimotor and cognitive function, reduced hippocampal neuronal cell loss, promoted angiogenesis and neurogenesis, and reduced neuroinflammation in a traumatic brain injury model [40]. On the other hand, Xu et al. reported that the BDNF-mediated MSCs exosome promotes neurogenesis and inhibits apoptosis in traumatic brain injury model rats [41].

Alzheimer’s disease, which is the most common type of dementia, is characterized by the degeneration and death of neurons in the basal forebrain, hippocampus, and cerebral cortex. Some reports demonstrate the efficacy of MSCs for the transgenic APP/PS1 mouse model, which is a mimic of Alzheimer’s disease. Positive effects have been reported by delivering BM-MSCs into the lateral ventricles of a transgenic APP/PS1/tau mouse model. Better preservation of working memory and downregulation of potentially toxic Aβ*56 levels in the entorhinal cortex have been described [42]. On the other hand, Routajangout et al. used UC-MSCs for APP/PS1 mouse by intracarotid injections. They reported a reduction of the cognitive loss and reduction of Aβ deposits in the cerebral cortex and the hippocampus [43].

There are also many basic research studies that investigate the efficacy of MSCs for Huntington’s disease, which is characterized by progressive neuronal cell death, resulting in cognitive decline, involuntary choreic movements, and psychiatric disturbances. Dey et al. evaluated the therapeutic effects of the transplantation of BM-MSCs genetically engineered to over-express BDNF or NGF on motor deficits and neurodegeneration in YAC 128 transgenic mice [44]. They demonstrated that YAC 128 mice that were transplanted with MSCs over-expressing BDNF had the longest latencies on the rotarod and the least amount of neuronal loss within the striatum of the YAC 128 mice. In addition, striatal injection of BM-MSCs engineered to overexpress BDNF has been reported to decrease striatal atrophy and reduce anxiety in YAC128 mice [45]. These genetically modified MSCs could potentially be a stem cell-based neurotherapeutics for neurodegenerative disorders.

## 3. Clinical Application of MSCs for Neurological Disorders

Based on the mechanisms suggested by the basic experiments mentioned above, several clinical trials using MSCs for neurological disorders have been conducted, and the recent clinical reports are summarized in this review.

Most of these clinical studies were performed with adult participants, while trials focusing on cerebral palsy were performed with children. Regarding the origin of MSCs; BM, UC, UCB, and AD sources have all been used. In addition, DPSCs are used for clinical trials for neurological disorders [46,47]. As for the administration of MSCs, most clinical studies adopt IV and/or IT. These clinical trials have mainly reported on the feasibility and efficacy of MSC therapies for neurological disorders, with some reporting adverse events, such as fever, vomiting, and headaches, while severe adverse events have not been observed.

### 3.1. Ischemic Stroke

The recent clinical reports using MSCs for the treatment of ischemic stroke are summarized in Table 1. Some studies have reported on the safety and feasibility of BM-MSCs in patients with ischemic stroke injury [48,49,50,51]. In these clinical trials, patients received intravenous injections of BM-MSCs, and an improvement in neurological functioning was observed, while no treatment-related adverse events were seen. Qiao et al. highlighted the safety and feasibility of the co-transplantation of neural stem/progenitor cells (NSPCs) and UC-MSCs in patients who had suffered from an ischemic stroke [52]. In the study, no tumorigenesis was found during a two-year follow-up, and the neurological functions, disability levels, and daily living abilities of the patients had improved. Jiang et al. reported on the safety and efficacy of UC-MSCs delivered via a catheter to a near-lesion site for treating an infarction in the middle cerebral artery territory [53]. UC-MSCs were infused via catheterization in the M1 segment of the middle cerebral artery. Cell delivery was performed successfully in all the patients, and no major accidents were observed. After this cellular therapy, two of the three ischemic stroke patients demonstrated improved muscle strength. These reports suggest that the transplantation of MSCs in subjects with ischemic stroke is safe and may promote neurological improvement. On the other hand, Nagpal et al. conducted a clinical trial using DPSC for stroke called TOOTH (The Open study of dental pulp stem cell Therapy in Humans) and are investigating the use of autologous stem cell therapy for stroke survivors with chronic disability [47].

### 3.2. Spinal Cord Injury

In most of the clinical trials involving MSC treatment for spinal cord injury (SCI), MSCs were administered via intrathecal or direct infusion to the injured lesion (Table 2). Vaquero et al. reported that patients administered BM-MSCs showed variable clinical improvements in sensitivity, motor power, spasms, spasticity, neuropathic pain, sexual function, and/or sphincter dysfunction, regardless of the level/degree of injury, age, or time elapsed since the SCI [54]. Hur showed the effects and safety of the intrathecal transplantation of autologous AD-MSCs in patients with SCI. Over the 8 months of follow-up, patients who received intrathecal transplantation of autologous AD-MSCs for SCI treatment did not experience any serious adverse events, and several patients showed mild improvements in neurological function [55]. Transplanting collagen scaffolds with human UC-MSCs has also been reported to have therapeutic potential as a treatment for SCI. Collagen scaffolds with human UC-MSCs were transplanted into the injury site directly, and the recovery of sensory and motor functions was observed in both patients [56]. Oh et al. reported on the injection of autologous BM-MSCs into the intramedullary area and subdural space and concluded that this single MSCs application was safe, but it had a very weak therapeutic effect compared with multiple MSC injections [57]. Therefore, further clinical trials to enhance the effect of MSCs are necessary in the future.

### 3.3. Cerebral Palsy

Recently, MSCs have been emerging for use in potential new therapeutic treatments for children with cerebral palsy. The recent clinical reports using MSCs for the treatment of cerebral palsy are summarized in Table 3. Huang et al. reported on a randomized, placebo-controlled trial of UCB-MSC infusion in children with cerebral palsy [65]. The infusion group was comprised of 27 patients, each of whom received four infusions of UCB-MSCs and basic rehabilitation treatment, whereas another 27 patients were assigned to the control group and received 0.9% normal saline and basic rehabilitation treatment. The changes in the gross motor and comprehensive functional scale in the UCB-MSC infusion group were significantly higher than those in control group at 3-, 6-, 12-, and 24-months post treatment. Liu et al. investigated whether BM-MSCs and BM-mononuclear cells (BM-MNCs) had any difference in curative effect regarding their use in the treatment of cerebral palsy. Their results indicated that BM-MSC transplantation for the treatment of cerebral palsy is safe and can improve gross and fine motor function significantly when compared with the results of BM-MNC treatment [66]. Cerebral palsy and its associated conditions can cause significant economic burdens to families. Therefore, clinical trials that may lead to new cell therapy strategies should be further investigated.

### 3.4. Amyotrophic Lateral Sclerosis (ALS)

ALS is a fatal neurodegenerative disease characterized by the degeneration of motor neurons in the brain and spinal cord, resulting in progressive muscle weakness and respiratory failure. The recent clinical reports using MSCs for the treatment of ALS are summarized in Table 4. Berry et al. highlighted the safety and efficacy of neurotrophic factor (NTF)-secreting MSCs (NurOwn^®^, autologous bone marrow-derived MSCs, induced to secrete NTFs) delivered by combined intrathecal and intramuscular administration to participants with ALS in a phase 2 randomized controlled trial [71]. The rate of disease progression (Revised ALS Functional Rating Scale (ALSFRS-R) slope change) in the overall study population was similar in the treated and placebo participants, while in a prespecified rapid progressor subgroup, the rate of disease progression improved at early time points. Furthermore, CSF neurotrophic factors increased, and associated inflammatory biomarker levels decreased in the treated participants post-NTF-secreting MSC transplantation. Another report showed that intrathecal and intramuscular administration of BM-MSC secreting neurotrophic factors in patients with ALS is safe and may provide clinical benefits [72]. Syková et al. demonstrated that the intrathecal application of BM-MSCs in ALS patients is a safe procedure and that this treatment could slow down the progression of the disease; a reduction in ALSFRS decline at three months after application was observed which, in some cases, persisted for six months [73]. Oh et al. reported that two repeated intrathecal injections of autologous BM-MSCs was a safe and feasible treatment for ALS patients throughout the duration of a 12-month follow-up period [74]. These results support the possibility that the use of MSCs in ALS patients could slow down the progression of the disease.

### 3.5. Multiple Sclerosis

Multiple sclerosis is a chronic immune-mediated inflammatory disease in which the immune system progressively destroys the myelin sheath in the central nervous system. This disease can last from a few months to many years. The recent clinical reports using MSCs for the treatment of multiple sclerosis are summarized in Table 5. Petrou et al. evaluated the optimal safe and effective clinical transplantation of MSCs in patients with active and progressive multiple sclerosis [79]. In the study, patients were randomized into three groups and treated intrathecally (IT) or intravenously (IV) with autologous BM-MSCs or sham injections. Significantly fewer patients experienced treatment failure in the MSC-IT and MSC-IV groups compared with those in the sham-treatment group. During the 1-year follow-up period, no evidence of disease activity was observed in 58.6% and 40.6% of patients treated with MSC-IT and MSC-IV, respectively, compared with 9.7% in the sham-treated group. MSC-IT transplantation induced additional benefits regarding the relapse rate, and the researchers concluded that the IT administration was more efficacious than the IV administration regarding several parameters of the disease. Furthermore, a safety and feasibility study was completed, focusing on the use of UC-MSCs for treating multiple sclerosis. Twenty subjects were enrolled in the study, and symptom improvements were most notable a month after treatment [80]. Infusion with MSCs is considered safe and feasible in patients with multiple sclerosis. However, larger studies investigating the number of doses and route of administration are needed to assess potential therapeutic benefits of this technique. 

### 3.6. Parkinson’s Disease

Parkinson’s disease is the common and progressive neurodegenerative disease with major symptoms such as bradykinesia, impaired posture, and tremor. Some studies have reported on the safety and feasibility of MSCs in patients with Parkinson’s disease (Table 6). Canesi et al. demonstrated the feasibility of BM-MSC in Parkinson’s disease patients. One year after cell infusion, all treated patients were alive, except one, who died 9 months after the infusion for reasons not related to cell administration or to disease progression (accidental fall), and in all treated patients, motor function rating scales remained stable for at least six months during the one-year follow-up [89]. On the other hand, Carstens et al. showed the efficacy of AD-MSCs in two patients with Parkinson’s disease. After the administration of AD-MSCs, subjective functional recovery after 2 weeks and up to 5 years are observed [90].

### 3.7. Traumatic Brain Injury

Traumatic brain injury is one of the major serious diseases that threaten human life and health, causing traffic accidents, collisions with hard objects, and falls from high places. With improving medical technology, the survival rate of patients with traumatic brain injury has increased significantly. However, the prognosis for patients with severe TBI remains poor, such as disturbance of consciousness and motor disorder. The recent clinical reports using MSCs for the treatment of traumatic brain injury are summarized in Table 7. Wang et al. showed the results of a phase 2 clinical trial using UC-MSCs for traumatic brain injury patients [91]. Forty patients with sequelae of traumatic brain injury were randomly assigned to the stem cell treatment group or the control group, and UC-MSCs administration improved the neurological function and self-care in patients after 6 months. On the other hand, Tian et al. explored the clinical therapeutic effects and safety of autologous BM-MSCs therapy for traumatic brain injury by lumbar puncture [92]. The results showed improvement in the function of brain in the form of post-therapeutic improvements in consciousness and motor functions. In addition, they showed the age of patients and the time elapsed between injury and therapy had effects on the outcomes of the cellular therapy, and no correlation was found between the number of cell injections and improvements.

## 4. Room for Improvement of MSCs Therapy and Future Perspectives

Many clinical trials using several sources of MSCs for neurological disorders have been conducted mentioned above. There are many basic research studies using MSCs for the Huntington’s disease model and Alzheimer’s disease model [93]; however, little clinical trials are reported for these diseases. Now, clinical trials using MSCs for Huntington’s disease and Alzheimer’s disease are ongoing (ClinicalTrials.gov Identifier: NCT03252535, NCT04388982, NCT04040348, NCT02833792); therefore, the feasibility and efficacy of MSCs for Huntington’s disease and Alzheimer’s disease are expected.

Regarding allogeneity, autologous transplantation is thought to be desirable when considering the possibility of rejection, but this depends on the sources of MSCs. It would be difficult to isolate autologous BM- or AD-MSCs in infants and children with cerebral palsy compared to autologous MSCs from UC and UCB, because BM and AD sampling involves invasive procedures. In contrast, collecting the autologous UC- and UCB-MSCs of adults is very difficult, because this would have required the cryopreservation of these cells decades ago. 

As for the administration route, many clinical trials have adopted intrathecal and intravenous administration, with some papers having reported a significant difference in efficacy depending on the route of administration. The number of administration events is also important as the therapeutic effectiveness of intrathecal administration of MSCs is reported to be related to the levels of neurotrophic factor and anti-inflammatory cytokines in ALS patients. Therefore, the potential therapeutic effect of a single treatment with MSCs would not be long-lasting, because these cells gradually disappear in cerebrospinal fluid over time, meaning that multiple MSC administration events would be needed to sustain the therapeutic effects [74,77]. On the other hand, no correlation between the number of cell injections and improvements is also reported [92]. There is another problem that results from multiple MSCs injection. Five participants received multiple MSCs injection developed new class I anti-human leukocyte antigen (HLA) antibodies, which are associated with a specific lot of UC-MSCs or with a partial HLA match between donor and recipient [94]. These antibodies were reported to be clinically silent and not associated with any clinical manifestations to date. Therefore, it is important to further investigate the appropriate protocol for the administration of MSCs considering the sources, route, and number of times they are administered.

The paracrine factors secreted from MSCs seem to exert therapeutic effects rather than the actual differentiation of themselves [95]. Unlike cell therapies, the administration of exosomes derived from MSCs seems to have no risks of adverse effects such as cellular rejection and thrombosis. Additionally, different sources of MSCs have different exosome characteristics; therefore, it is necessary to select appropriate exosomes that contain enough neurotrophic factors such as BDNF, NGF, and HGF. Developing exosomes secreted from MSCs for clinical use have several challenges such as reliability, reproducibility, and robust techniques to isolate and purify therapeutic exosomes and to produce exosomes on a large scale with good manufacturing practices standards for clinical use [17]. Shekari et al. identified gaps in the current method of information gathering by systematically reviewing a substantial number of publications. They showed a lack of standards and poor consensus on different aspects of isolation processes; quantifications and tests of purity were important problems in the exosome-based therapies that made it difficult to compare reports [96]. Therefore, the translation of MSCs and exosomes of MSCs from the preclinical to clinical level presents several challenges to investigators and clinicians. 

International large clinical trials using the same products for neurological disorders will be needed to establish suitable therapeutic protocol for the clinical use of MSCs and exosomes in the future.

## 5. Conclusions

**Figure 2:**
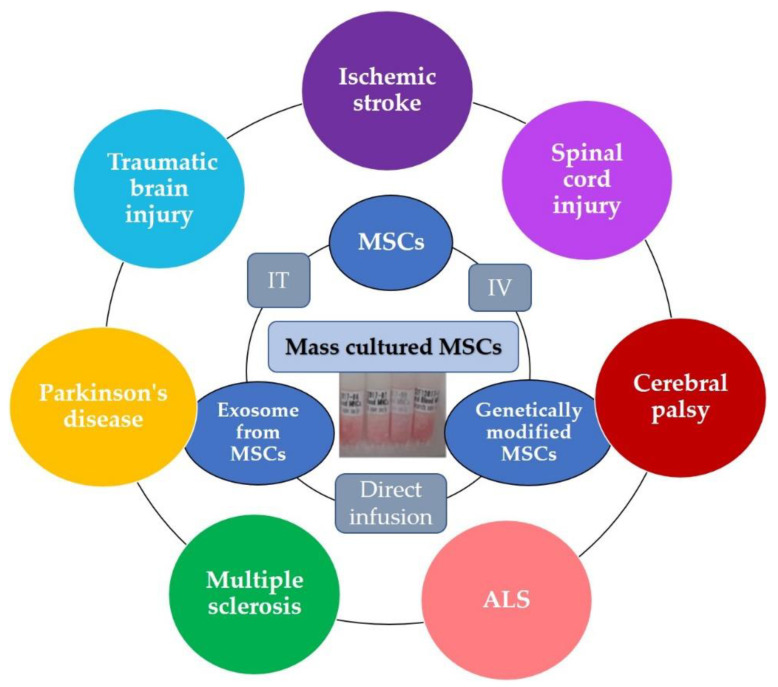
Therapeutic protocol of the clinical use of MSCs and exosomes for neurological disorders in the future.

Many existing therapies are insufficient in some cases for neurological disorders. Therefore, new alternative therapeutics are expected for the treatment of these neurological disorders. Recent clinical trials indicate that the use of MSCs as a new cell therapy is expected to be effective in combination with conventional rehabilitation and other medication. Furthermore, some clinical trials have been demonstrated the efficacy of exosome derived from MSCs and genetically modified neurotrophic factor-secreting MSCs for neurological disorders, which is a treatment method that can be expected to be very effective in the future (Figure 2).

The best-established protocol of MSCs therapy does not yet exist; however, many sources and many protocols of clinical trials will expand the potentials of the treatable area in neurological disorders and lead to established therapies. Further large clinical studies on using MSCs to treat neurological diseases will extend our knowledge of these cells in the future.

## Figures and Tables

**Table 1:** Summary of recent clinical trials using MSCs for ischemic stroke.

Reference	Disease	Source	Number		Mean Age (Range), Year	Route of Administration	Number of Cells	Number of Treatments	Results	Adverse Events
			Trial	Control						
Chung et al. [48]	Ischemic stroke (Phase 3)	BM	39	15	68 (28–83)	IV	1 × 10^6^/kg	1	Lower extremity motor functional recovery after 3 months	No
Bhasin et al. [49]	Ischemic stroke	BM	6	6	42.8 (20–60)	IV	5–6 × 10^7^ cells	1	Improvement in the activities of daily living (ADL) after 156 and 208 weeks	No
Qiao et al. [52]	Ischemic stroke (Phase 1/2)	UC	6	0	56.17 (3–85)	IVIV + IC	IV:MSC 0.5 × 10^6^/kg IC:MSC 5 × 10^6^ cells NSPC 6 × 10^6^ cells at one-week interval	4 or 1 + 3	Improvement in the neurological functions and ADL after 3, 12, 24 months	Fever, dizziness
Jiang et al. [53]	Ischemic and hemorrhagic stroke	UC	4	0	40–59	IA (intra-arterial) via catheterization	2 × 10^7^ cells	1	Motor functional recovery and improvement in the ADL after 3 and 6 months	No
Bhasin et al. [50]	Ischemic stroke	BM	20 MSC6 MNC14	20	45.1	IV	5–6 × 10^7^ cells	1	Improvement in the ADL after 8 and 24 weeks	No
Honmou et al. [51]	Ischemic stroke (Phase 1)	BM	12	0	59.2 (41–73)	IV	0.6–1.8 × 10^8^ cells	1	Incremental daily rate of change in the disability scales during 12 months	Fever, nausea, itching

IV, intravenous injection; IT, intrathecal injection; IC, intracranial; BM, bone marrow; UC, umbilical cord.

**Table 2:** Summary of recent clinical trials using MSCs for SCI.

Reference	Disease	Source	Number		Mean Age (Range), Year	Route of Administration	Number of Cells	Number of Treatments	Results	Adverse Events
			Trial	Control						
Xiao et al. [56]	Spinal cord injury (Phase 1)	UCB	2	0	28, 30	Transplantation into the lesion with collagen scaffolds	4 × 10^7^ cells	1	Motor functional recovery after 3, 6, 12 months Sensory functional recovery after 2, 4, 12 months	No
Vaquero et al. [54]	Spinal cord injury (Phase 2)	BM	11	0	44.91 (28–62)	IT	100 × 10^6^ cells at 3 months interval	3	Motor, sensory and bladder–bowel functional recovery after 4, 7, 10 months	No
Vaquero et al. [58]	Post-traumatic syringomyelia (Phase 2)	BM	6	0	39 (30–50)	Direct injection into the lesion	300 × 10^6^ cells	1	Achieving reduction of syrinx and valiable clinical improvements after 6 months	No
Vaquero et al. [59]	Spinal cord injury (Phase 2)	BM	10	0	42.2 (34–59)	IT	30 × 10^6^ cells at 3-months interval	4	Motor, sensory and bladder–bowel functional recovery after 3, 6, 9, 12 months	Headache, puncture pain
Satti et al. [60]	Spinal cord injury (Phase 1)	BM	9	0	31.6 (24–38)	IT	1.2 × 10^6^/kg at 4 weeks interval	2 or 3	Only safety assessment	No
Oh et al. [57]	Spinal cord injury (Phase 3)	BM	16	0	40.9 (18–65)	Direct injection into the lesion + IT	1.6 × 10^7^ cells 3.2 × 10^7^ cells	1	Very weak therapeutic efficacy after 6 months	Sensory deterioration, muscle rigidity, tingling sense
Hur et al. [55]	Spinal cord injury (Phase 1)	AD	14	0	41.9 (20–66)	IT	3 × 10^7^ at 1-month interval	3	Motor and sensory functional recovery after 8 months	Nausea, vomit, headache
Mendonça et al. [61]	Spinal cord injury (Phase 1)	BM	14	0	35.7 (23–61)	Direct injection into the lesion	5 × 10^6^ cells/cm^3^ per lesion volume	1	Motor, sensory, and bladder–bowel functional recovery after 6 months	Low-intensity pain at the incision site, cerebrospinal fluid leak
Cheng et al. [62]	Spinal cord injury (Phase 2)	UC	10	34	35.3 (19–57)	Direct injection into the lesion	2 × 10^7^ cells at 10 days interval	2	Motor, sensory, and bladder functional recovery after 6 months Superior efficacy than that of rehabilitation therapy	Radiating neuralgia
Dai et al. [63]	Spinal cord injury (Phase 1/2)	BM	20	20	22–54	Direct injection into the lesion	20 × 10^6^ cells	1	Motor, sensory, and bladder functional recovery after 6 months	Fever, headache, pain
Karamouzian et al. [64]	Spinal cord injury (Phase 1/2)	BM	11	20	33.2 (23–48)	IT	0.7–1.2 × 10^6^ cells	1	Possible efficacy in the motor and sensory function	No

IT, intrathecal injection; BM, bone marrow; AD, adipose; UC, umbilical cord; UCB, umbilical cord blood.

**Table 3:** Summary of recent clinical trials using MSCs for cerebral palsy.

Reference	Disease	Source	Number		Mean Age (Range), Year	Route of Administration	Number of Cells	Number of Treatments	Results	Adverse Events
			Trial	Control						
Gu et al. [67]	Cerebral palsy (Phase 1/2)	UC	19	20	4.29	IV	4.5–5.5 × 10^7^ cells at 7-day intervals	4	Gross motor and comprehensive functional recovery and improvement in the ADL after 3, 6, 12 months	No
Ahn et al. [68]	Intraventricular hemorrhage (Phase 1)	UCB	9	0	11.6 (7–15) (days)	Intraventricular	5 × 10^6^/kg or 1 × 10^7^/kg	1	Only safety assessment	No
Huang et al. [65]	Cerebral palsy (Phase 1/2)	UCB	27	27	7.4 (3–12)	IV	5 × 10^7^ cells at 7-day intervals	4	Gross motor and comprehensive functional recovery after 3, 6, 12, 24 months	No
Liu et al. [66]	Cerebral palsy (Phase 1/2)	BM	MSC 33 MNC34	35	7–132 (months)	IT	1 × 10^6^/kg at 3–4-day intervals	4	Motor functional recovery after 12 months	No
Wang et al. [69]	Cerebral palsy (Phase 4)	UC	16 (8 twins)	0	6.29 (3–12)	IT	1–2 × 10^6^ cells at 3–5-day intervals	4	Motor functional recovery after 1 and 6 months	No
Wang X et al. [70]	Cerebral palsy	BM	46	0	6–180 (months)	IT Intra-Parenchymal	2 × 10^7^ cells 4 × 10^7^ cells at 5-day intervals	2 + 1 or 4	Gross motor functional recovery after 1, 6, 18 months	No

IV, intravenous injection; IT, intrathecal injection; BM, bone marrow; UC, umbilical cord; UCB, umbilical cord blood.

**Table 4:** Summary of recent clinical trials using MSCs for ALS.

Reference	Disease	Source	Number		Mean Age (Range), Year	Route of Administration	Number of Cells	Number of Treatments	Results	Adverse Events
			Trial	Control						
Berry et al. [71]	ALS (Phase 2)	BM-NTF	36	12	51.1 (26–71)	IM + IT	IM: 48 × 10^6^ cells IT: 125 × 10^6^ cells	1	Improvement in the rate of disease progression after 6 months	Headache, fever, back pain, injection site bruising
Syková et al. [73]	ALS (Phase 1/2)	BM	26	0	51.2 (33–64)	IT	15 ± 4.5 × 10^6^ cells	1	Slowing down of the diseaseprogression after 3, 6, 9 months	Headache
Staff et al. [75]	ALS (Phase 1)	AD	27	0	36–75	IT	1 × 10^7^, 5 × 10^7^, 5 × 10^7^ × 2, 1 × 10^8^, 1 × 10^8^ × 2	1 or 2	Only safety assessment	Temporary back and leg pain in the highest dose
Petrou et al. [72]	ALS (Phase 1/2)	BM-NTF	26	0	48.1, 50.8 (23–65)	IM IT IM + IT	IM: 2.4–4.8 × 10^7^ cells IT: 1.0–2.0 × 10^6^ /kg	1	Improvement in the rate of disease progression after 6 months	Fever, vomiting, headache
Rushkevich et al. [76]	ALS	BM-MSC and neural induced MSC	10	15	54.5, 55.0 (37–66)	IV + IT	0.5–1.5 × 10^6^/kg 5.0–9.7 × 10^6^ cells at 5–7-month intervals	1 or 2	Slowing down of the disease progression after 12 months	Fever, headache
Oh et al. [74]	ALS (Phase 1)	BM	8	0	45.7 (29–62)	IT	1 × 10^6^/kg at 26-day intervals	2	No efficacy after 6 months	Fever, pain, headache
Kim et al. [77]	ALS	BM	37	0	52.7, 48.8	IT	1 × 10^6^/kg at one-month intervals	2	Trophic factors associated with a positive response to treat	No
Mazzini et al. [78]	ALS (Phase 1)	BM	19	0	20–75	Direct injection into spinal cord	7–152 × 10^6^ cells	1	No long-term adverse effect after nearly 9 years	No

IV, intravenous injection; IT, intrathecal injection; IM, intramuscular injection; BM, bone marrow; AD, adipose.

**Table 5:** Summary of recent clinical trials using MSCs for multiple sclerosis.

Reference	Disease	Source	Number		Mean Age (Range), Year	Route of Administration	Number of Cells	Number of Treatments	Results	Adverse Events
			Trial	Control						
Petrou et al. [79]	Multiple sclerosis (Phase 2)	BM	16, 16	16	47.6 (37.9–57.3)	IT or IV	1 × 10^6^/kg at 6-month intervals	1 or 2	Improvement in the course of the disease and comprehensive functional recovery after 3, 6, 12 months. IT is more efficacious than IV	No
Fernández et al. [81]	Multiple sclerosis (phase 1/2)	AD	10, 9	11	44.8 47.8 46.3	IV	1 × 10^6^/kg or 4 × 10^6^/kg	1	Partial efficacy in the imaging studies and evoked potentials after 12 months	urinary infection
Riordan et al. [80]	Multiple sclerosis (phase 1/2)	UC	20	0	41.15	IV	20 × 10^6^ cells at 1–4-day intervals	7	Comprehensive functional recovery after one month	Headache, fatigue
Harris et al. [82]	Multiple sclerosis (Phase 1)	BM MSC -derived neural progenitors	20	0	27–65	IT	5.3–10 × 10^6^ cells at 3-month intervals	3	Motor, bladder and comprehensive functional recovery after 3 months	headache, fever
Dahbour et al. [83]	Multiple sclerosis (Phase 1/2)	BM MSC-CM	10	0	34.9 (18–54)	IT	93–168 × 10^6^ cells CM:13–20 mL at 1-month intervals	1 + 1	Comprehensive functional recovery after 12 months	Pain, headache, fever
Llufriu et al. [84]	Multiple sclerosis (Phase 2)	BM	9	0	36.8 (23–48)	IV	1–2 × 10^6^/kg	1	Improvement in the imaging studies after 6 months	No
Li et al. [85]	Multiple sclerosis	UC	13	10	41.7, 39.4	IV	4 × 10^6^ cells/kg at 2-week intervals	3	Improvement in the overall symptoms and fewer incidences of relapse during 12 months	No
Bonab et al. [86]	Multiple sclerosis (Phase 2)	BM	25	0	34.7 (23–50)	IT	2.95 × 10^7^ cells	1	Improvement or stabilization in the course of the disease during 12 months	Fever, nausea, weakness in the lower limbs, headache
Lee et al. [87]	Multiple sclerosis (Phase 2)	BM	16	17	56.1, 55.8	IA (intra-arterial) + IV	IA: 4 × 10^7^ cells IV: 4 × 10^7^ cells at 30-day intervals	1 + 3	Efficacy in preventing the progression of neurological deficits during 12 months	Small ischemic lesions
Connick et al. [88]	Multiple sclerosis (Phase 2)	BM	10	0	48.8 (40–53)	IV	1.6 × 10^6^/kg	1	Visual functional recovery after 10 months	Macular rash, self-limiting infections

IV, intravenous injection; IT, intrathecal injection; BM, bone marrow; AD, adipose; UC, umbilical cord.

**Table 6:** Summary of recent clinical trials using MSCs for Parkinson’s disease.

Reference	Disease	Source	Number		Mean Age (Range), Year	Route of Administration	Number of Cells	Number of Treatments	Results	Adverse Events
			Trial	Control						
Canesi et al. [89]	Progressive supranuclear palsy (Phase 1)	BM	5	0	60–68	IA (intra-arterial) via catheterization	1.7 (1.2–2.0) × 10^6^/kg	1	Clinical stabilization for at least 6 months during the one-year follow-up	Transient left hemiparesis
Carstens et al. [90]	Parkinson’s disease (Case studies)	AD MSC-derived stromal vascular fraction	2	0	72, 50	Facial and nasal transplantation	6.0 × 10^7^ cells	1	Subjective functional recovery after 2 weeks and up to 5 years	No

BM, bone marrow; AD, adipose.

**Table 7:** Summary of recent clinical trials using MSCs for traumatic brain injury.

Reference	Disease	Source	Number		Mean Age (Range), Year	Route of Administration	Number of Cells	Number of Treatments	Results	Adverse Events
			Trial	Control						
Wang et al. [91]	Traumatic brain injury (Phase 2)	UC	20	20	27.5 ± 9.4 28.6 ± 10.1	IT	6.0 × 10^7^ cells	4	Comprehensive functional recovery and improvement in the ADL after 6 months	Mild dizziness, headache
Tian et al. [92]	Traumatic brain injury	BM	97	0	-	IT	3.0–5.0 × 10^6^ cells	1	Improvement of consciousness and motor function after 14 days	No

IT, intrathecal injection; BM, bone marrow; UC, Umbilical cord.
